# Supplementary material for: Genetic buffering and potentiation in metabolism
Source: PLoS Comput Biol. 2020 Sep 14;16(9):e1008185. doi: 10.1371/journal.pcbi.1008185 (PMC7514045; doi:10.1371/journal.pcbi.1008185)
Supplement: S1 Note — (PDF) [file pcbi.1008185.s001.pdf]

Genetic buffering and potentiation in metabolism

Juan F. Poyatos[1,2]

[1] Logic of Genomic Systems Lab (CNB-CSIC), Madrid 28049, Spain
[2] Center for Genomics and Systems Biology (NYU), New York 10003, USA

Supplementary Note 1.

The analyses in the main text show how the very same metabolic mutations could lead to different phenotypic variability (growth rate as the phenotype) depending on the presence or absence of a particular enzyme. This could imply that the presence of a particular mutation can alter the consequence of subsequent mutations in the growth rate of cancer cells. There is currently no available data to properly test this hypothesis. In this note, inspired by the original work of modifiers in genetic networks (Bergman and Siegal 2003) , we alternatively examine the effect of particular oncogenic mutations in the variability of an alternative phenotype: gene expression of metabolic enzymes.

We used a collection of 22 tumor types compiled by Hu et al. (Hu et al. 2013), which includes for each type a set of control and tumor samples. We asked whether the mutations acquired in specific tumors change the expression variability of the enzymes when compared to the control samples (no mutations). This would indicate that some of these mutations involve modifiers of gene expression variability.

We specifically examined a list of metabolic genes obtained from the Human metabolic reconstruction model (Swainston et al. 2016). Each dataset was normalized using the RMA algorithm in the Bioconductor 3. 7 packages (www.bioconductor.org) running under R version 3.7. To obtain S1 Fig, we quantified, for each enzyme, the standard deviation of expression in the corresponding set of control and tumor samples. We then calculated the fraction of enzymes that present more variability in the tumor than in the control (this defines the "ratio of enzymes with more variability in tumor conditions") and also calculated a null for this ratio by randomizing the expression values between control and tumor samples (to compute a new ratio, 1000 randomizations). Most tumors led to an increase in variability.

We acknowledge, however, that this variability could be caused extrinsically as tumor microenvironments are typically heterogeneous. If the variability in expression were to be dominantly caused by extrinsic forces, this ratio would be mostly within a relatively narrow range of large values. But this does not seem to be the case, with ratios ranging from 0.1 to 0.9 and related tumors (or equivalent tumors in different experiments) exhibiting different ratios. Conservatively, this analysis shows that the variability of enzymes expression for the same tumor is influenced by the specific physiological conditions and genetic mutations of each individual tumor.

References

Bergman, Aviv, and Mark L. Siegal. 2003. ‘Evolutionary Capacitance as a General Feature of Complex Gene Networks’. *Nature* 424 (6948): 549–52. https://doi.org/10.1038/nature01765.

Hu, Jie, Jason W. Locasale, Jason H. Bielas, Jacintha O’Sullivan, Kieran Sheahan, Lewis C. Cantley, Matthew G. Vander Heiden, and Dennis Vitkup. 2013. ‘Heterogeneity of Tumor-Induced Gene Expression Changes in the Human Metabolic Network’. *Nature Biotechnology* 31 (6): 522–29. https://doi.org/10.1038/nbt.2530.

Swainston, Neil, Kieran Smallbone, Hooman Hefzi, Paul D. Dobson, Judy Brewer, Michael Hanscho, Daniel C. Zielinski, et al. 2016. ‘Recon 2.2: From Reconstruction to Model of Human Metabolism’. *Metabolomics* 12. https://doi.org/10.1007/s11306-016-1051-4.
