# Supplementary figures and images for: Genetic buffering and potentiation in metabolism
Source: PLoS Comput Biol. 2020 Sep 14;16(9):e1008185. doi: 10.1371/journal.pcbi.1008185 (PMC7514045; doi:10.1371/journal.pcbi.1008185)

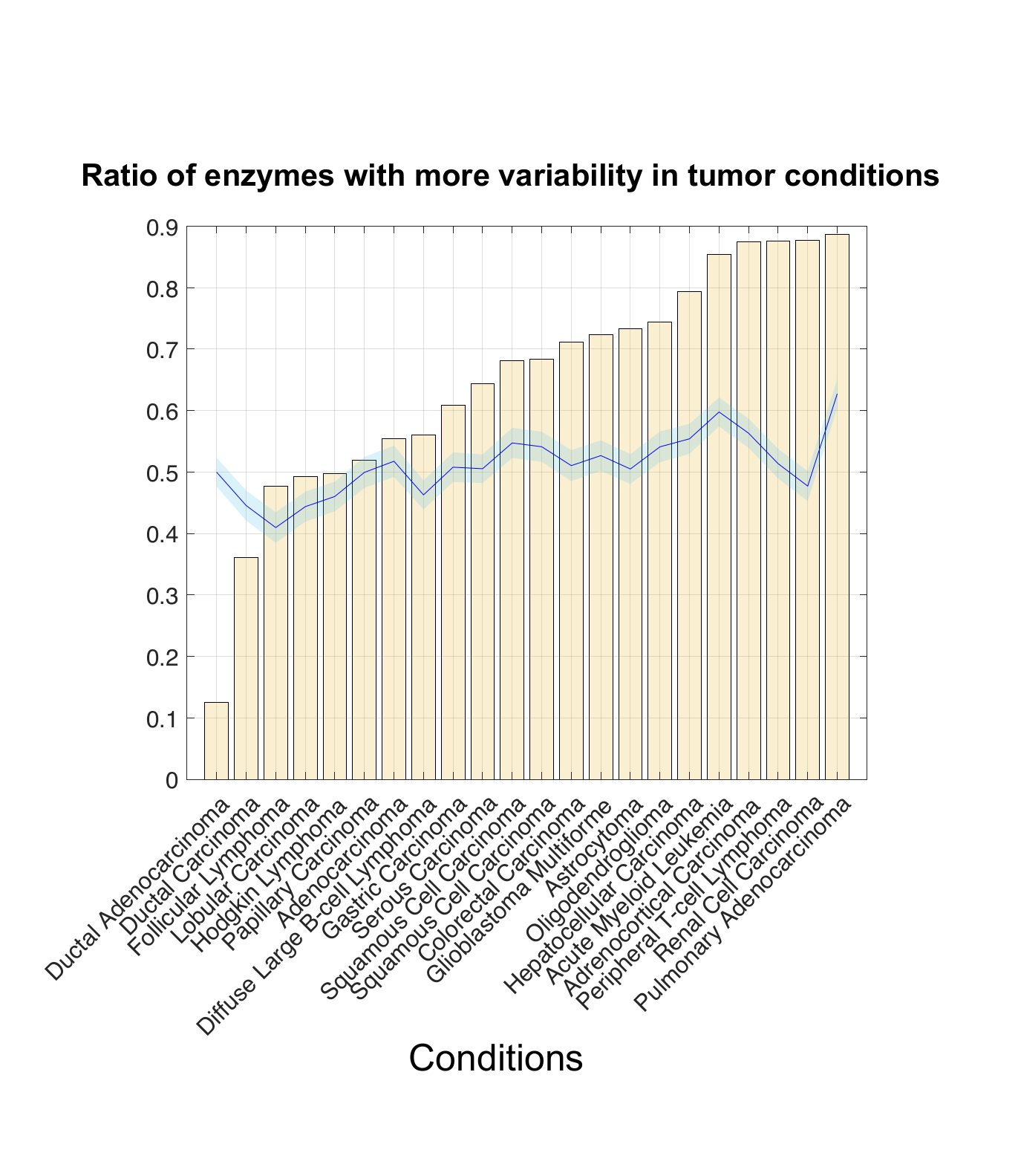

Supplement: S1 Fig — We used gene expression data of pairs of control and tumor samples to quantify the variability (standard deviation) in the expression of metabolic genes within each sample (see S1 Note for details). With these scores, we estimated the fraction of enzymes with more variation within the tumor sample than the control (this ratio is indicated by the orange bars, in increasing order). We also computed the expected null value of this score by randomization of expression data between tissue and control. We plot the mean null value of these randomizations (blue curve) and the +/- 2 std (blue shading, 1000 randomizations). (TIF) [file pcbi.1008185.s002.tif]
