# Supplementary material for: Genetic buffering and potentiation in metabolism
Source: PLoS Comput Biol. 2020 Sep 14;16(9):e1008185. doi: 10.1371/journal.pcbi.1008185 (PMC7514045; doi:10.1371/journal.pcbi.1008185)
Supplement: Attachment — Submitted filename: Poyatos_JF_response_to_reviewers.pdf [file pcbi.1008185.s008.pdf]

MINISTERIO
DE CIENCIA, INNOVACIÓN Y
UNIVERSIDADES

CSIC
Consejo Superior de Investigaciones Científicas

Logic of Genomic Systems Laboratory
Spanish National Biotechnology Centre CNB

Reviewer #1:

>Minor Grammatical errors.
The manuscript has been significantly edited to improve its readability.

>If the authors can validate the claims by existing biological (wet lab data) in this study it would be better. As there seems to be no validation.

To confirm our predictions, one would need to develop a set of mutation lines where some mutations that modify metabolic flux accumulate. One should additionally quantify the growth rate, as a complex phenotype in these lines. These measurements had to be done in different genetic backgrounds, i.e., wild-type and others, corresponding to single-enzyme deletions. This combined work is currently being discussed in my laboratory but is clearly beyond the scope of this manuscript.

Moreover, we have incorporated a brief analysis of gene expression variability in cancer and control samples to further illustrate our final comments on the implications of this study for the understanding of tumor progression. See Supplement.

>1000 gene mutation data should be provided
Note that there are 10000, rather than 1000, random mutations in the fluxes and that this set only corresponds to the YPD environment. That means that we had to include too many lists (of 10000 mutations). To better understand these sets, we decided to discuss the type of mutations whose impact on growth rate changed as a function of the background (section "metabolic rationale underlying buffers and potentiators").

We would like to further clarify to the reviewer the context of this work. The traditional argumentation behind genetic modifiers stated the existence of some specific molecular elements able to alter the impact of mutations in genetic systems. This approach was prompted by the experiments of Hsp90 (and a few other molecular elements) and thus pervaded in the literature. Later studies with toy computational network models modified this picture and proposed that most elements of genetic systems could act as modifiers. Buffering and potentiation can result from *interactions* and should be ubiquitous across biological networks. This computational result was initially corroborated with some examination of gene expression variability and, more recently, with morphometric analysis. Notably, one of the main requirements in the experimental investigation of this issue is the requisite for large-scale quantification of phenotypes in both wild-type and mutant systems. There is a great need for this type of experiment.

jpoyatos@cnb.csic.es

c/ Darwin, 3
Campus de Cantoblanco
28049 MADRID
TEL.: 91 585 5309
FAX: 91 585 4506

MINISTERIO
DE CIENCIA, INNOVACIÓN Y
UNIVERSIDADES

CSIC
Consejo Superior de Investigaciones Científicas

Logic of Genomic Systems Laboratory
Spanish National Biotechnology Centre CNB

Give these limitations, one of the original contributions of this work is to propose a computational system where large-scale quantification of complex phenotypes can be simulated, for the study of genetic modifiers. Genome-scale metabolic models satisfy this condition with the added feature of not being a toy model. Instead, these metabolic models have become a standard in Systems Biology for studying the consequences of metabolic perturbations on cellular function with a great competence for prediction. Among other contributions, they have been responsible for the discovery of new antibiotics and chemotherapeutics, the design of bacterial strains optimized for industrial production of substances of interest, and the better comprehension of human metabolic diseases (Burgard, Pharkya, and Maranas 2003; Pagliarini et al. 2016; Raman, Vashisht, and Chandra 2009).

It is also important to understand that beyond the discussion of some of the specific predictions, this work demonstrates for the first time the role of enzymes as genetic modifiers in a metabolic model that fully account for the structure and function of extant metabolisms.

We thank the reviewer for his/her comment and hope that he/she considers now this manuscript suitable for publication.

jpoyatos@cnb.csic.es

c/ Darwin, 3
Campus de Cantoblanco
28049 MADRID
TEL.: 91 585 5309
FAX: 91 585 4506

Reviewer #2:

>1, The author may want to introduce more on what the metabolic model is and how it fits in the simulation of evolutionary studies.

We now described metabolic models in some more detail and added a bibliographic item explaining flux balance analysis.

>2, Is there any reason why to choose iND750 model as the main model for simulation instead of a more recent metabolic model which contains more reactions and genes?

We mostly worked with *Saccharomyces cerevisiae* iND750 because this enables us to incorporate all necessary complexity from yeast metabolism, has been empirically corroborated, and moderates the *substantial* computational load associated with our analysis. Note, however, that we also examined a more complex *Saccharomyces cerevisiae* model (iAZ900 in YPD medium) to validate the general appearance of buffers and potentiators in metabolism (see Table S3 and description of the models in Materials and Methods).

>3, The author may want to specify the setting of lower and upper bound of flux for those unlimited reactions compare to the limited flux. And please provide the Matlab code in how the simulation is done that can be posted on the journal website as supplementary material to increase the impact of the paper.

We are now including a core Matlab script describing how simulations are done, with all settings of flux bounds included.

>4, In the results part the author discussed the potential buffers and potentiators that was identified and its properties. Is there any corroborating experimental evidence shows that these enzymes working as buffers or potentiators? Is it possible to verify the findings?

To confirm our predictions, one would need to develop a set of mutation accumulation lines where some mutations that modify flux accumulate. One should additionally quantify the growth rate, as a complex phenotype in these lines. These measurements have to be done in different genetic backgrounds, i.e., wild-type and others corresponding to single-enzyme deletions. This combined work is currently being discussed in my laboratory but is clearly beyond the scope of this manuscript.

We consider, however, that the demonstration that enzymes can act as genetic modifiers whose specific action (buffer or potentiation) depends on the working of metabolism is a solid one, considering that genomic reconstructions are a valid representation of extant metabolisms. Note also that some of the predictions solely done with these models have been later corroborated experimentally. Among other contributions, they have been responsible for the discovery of new antibiotics and chemotherapeutics, the design of bacterial strains optimized for industrial production of substances of interest, and the better comprehension of human metabolic diseases (Burgard, Pharkya, and Maranas 2003; Pagliarini et al. 2016; Raman, Vashisht, and Chandra 2009).

jpoyatos@cnb.csic.es

c/ Darwin, 3
Campus de Cantoblanco
28049 MADRID
TEL.: 91 585 5309
FAX: 91 585 4506

MINISTERIO
DE CIENCIA, INNOVACIÓN Y
UNIVERSIDADES

CSIC
CONSEJO SUPERIOR DE INVESTIGACIONES CIENTÍFICAS

Logic of Genomic Systems Laboratory
Spanish National Biotechnology Centre CNB

>5, The author mentioned that FBA and MOMA have different objective functions. The model implements enzyme knockout by constrain all the flux of the reactions that the enzyme involved in the model to zero which is a similar approach the author construct MA lines. Wouldn't it be more consistency to use only FBA or MOMA to calculate the variability of growth rate in different simulations?

Note first that fluxes are not necessarily equal to zero in the MA lines. Beyond this, we interpreted each background as a "stable" metabolism, in which there has been enough time to optimize fitness after the enzyme deletion (as computed by FBA). For each background, we then analyzed flux alterations in the MA lines as perturbations on such reference metabolism. In this case, the optimized growth rate is the one that minimizes flux balance changes (as computed by MOMA). One could explore, of course, other means to implement mutations, but this does not invalidate our approach and the results we found.

>6, The discussion on how this work can contribute to cancer study seems conceptual and theoretical. The author may want to make changes on the last two paragraphs to make it easy to follow.

We have edited this paragraph. We have also incorporated a brief analysis of gene expression variability in cancer and control samples to further illustrate these final comments on the implications of this study for the understanding of tumor progression. See Supplement.

>7, Page 8 line 247 -250, The sentence "Besides, and while there is a general tendency to exhibit more buffering that potentiation, there exist situations in which potentiation is dominant and others in which the number of enzymes acting as buffers is severely reduced." seems confusing. Does the author mean "Besides, and while there is a general tendency to exhibit more buffering than potentiation, …"?

This has been corrected.

>8, Page 17 line 541, there is two "e.g." In the sentence. The author may want to carefully review the manuscript to avoid these minor mistakes.

This has been corrected.

We thank the reviewer for his/her comment and hope that he/she considers now this manuscript suitable for publication.

jpoyatos@cnb.csic.es

c/ Darwin, 3
Campus de Cantoblanco
28049 MADRID
TEL.: 91 585 5309
FAX: 91 585 4506
