# Supplementary material for: Genetic buffering and potentiation in metabolism
Source: PLoS Comput Biol. 2020 Sep 14;16(9):e1008185. doi: 10.1371/journal.pcbi.1008185 (PMC7514045; doi:10.1371/journal.pcbi.1008185)
Supplement: Attachment — Submitted filename: Poyatos_JF_response_to_reviewers.pdf [file pcbi.1008185.s009.pdf]

MINISTERIO
DE CIENCIA, INNOVACIÓN Y
UNIVERSIDADES

CSIC
Consejo Superior de Investigaciones Científicas

Logic of Genomic Systems Laboratory
Spanish National Biotechnology Centre CNB

Reviewer #1:

>The lists for all 10000 mutations need to be provided as supplementary files.
10000 gene mutation data lists are now provided (new Table S4).

>The corroboration of the study with cancer is still not completely elaborated. The work is linked to cancer just in the discussion, it js a well studied phenomenon that driver mutations propagate other mutations and all this depends on the type of cancer, onset, age and many other factors. It is felt that the work is forced linked to cancer. There is no link to the environments mentioned, to the genes mentioned just that tumor micro environment. The Supplementary note 1 is inadequate. It is already tumour environments are heterogeneous and hence there is going to gene expression variability, hence the enzymes, variability. What is the link to your work is not well established.

The connection with cancer is a corollary of the main result discussed throughout the manuscript. However, it appears that it was not argued well enough, given this comment. I will try to elaborate on this relation in the following by addressing each of the reviewer's remarks:

>> *it js a well studied phenomenon that driver mutations propagate other mutations and all this depends on the type of cancer, onset, age and many other factors*
I agree with this; these *other mutations* are typically known as *passenger* mutations (those that don't contribute to the development of cancer but that has occurred during its growth) and, as the referee argues, this process depends on many elements. Note that I never argued otherwise the in the manuscript. For instance, I commented on the discussion "how recent work already hinted, however, to more context-specific results where the tissue of origin, or cell lineage, etc. alters the metabolic adjustments created by the same mutation".

>> *It is felt that the work is forced linked to cancer.*
The essential theme of this work is to demonstrate, for the first time, that enzymes can work as buffers or potentiators of phenotypic variability. At its core, this implies that by altering a specific enzyme the range of impact of successive mutations in fitness (as the complex phenotype) becomes transformed. This implies that the very same mutational trajectory, with or without the modification of an enzyme, would cause *different* fitness diversity. This result has important consequences in cancer progression. A mutation of an enzyme does not only rewire metabolism in cancerous cells, as it is now largely studied, but modifies the impact of successive mutations and the diversity observed in the population. Most importantly, by inactivating an enzyme as part of a therapy, we could be altering the phenotypic effects of mutations and therefore tumor progression itself. A comparable effect was recently discussed with Hsp90 (see discussion in Geiler-Samerotte et al; doi: 0.1371/ journal.pbio.2000465), again another molecule that could act as potential target for therapy.

>> *The Supplementary note 1 is inadequate. It is already tumour environments are heterogeneous and hence there is going to gene expression variability, hence the enzymes, variability. What is the link to your work is not well established.*

As response to the comments of both referees in the previous round of reports, I tried to expand my discussion on the implications of these ideas in cancer with the use of available data. Given that the precise combination of mutation accumulation lines and fitness measurements –required for the validation of these ideas– are not available, I considered a complementary analysis with gene expression as phenotype.

jpoyatos@cnb.csic.es

c/ Darwin, 3
Campus de Cantoblanco
28049 MADRID
TEL.: 91 585 5309
FAX: 91 585 4506

The focus is still to appreciate how the presence/absence of a particular mutation(s) could be influencing phenotypic variability. I thus followed the original work on modifiers (Bergam and Siegal 2003). These authors used available data in *Saccharomyces cerevisiae*, where genome-wide expression had been assayed in a large collection of knockout strains (in which a single gene has been deleted) to quantify if gene expression variability was modified when one gene is knocked out. In my case, I tried to quantify if the presence of mutations associated with specific cancers modifies the expression variability of metabolic enzymes.

Available data comprises two types of samples: control, and tumor. Variability refers here to variability within each type of sample. I did this for 22 different classes of tumors to obtain a ratio of the number of enzymes that showed more variability in the tumor samples as compared to the corresponding control. If the variability in expression were to be dominantly caused by extrinsic forces, as the referee correctly argues tumor microenvironments are typically heterogeneous, this ratio would be mostly within a relatively narrow range of large values. But this does not seem to be the case, with ratios ranging from 0.1 to 0.9 and related tumors (or equivalent tumors in different experiments) exhibiting different ratios. Conservatively, this analysis shows that the variability of enzymes expression for the same tumor is influenced by the specific physiological conditions and genetic mutations of each individual tumor. I have incorporated an edited version of this *supplementary* note in the new version. Again, this is an effort to describe some of the implications of the main result, not the main result.

> If the researchers cannot do the mutational analysis to validate their claims they need to find existing research works that help in validation. Line 160 - 168 it is mentioned that 100 such lines (ref 21) have been generated, do the results of these 100 match your work? Does your work complement the ref stated?

Citation of this reference only indicates that accessible mutation accumulation lines (in different organisms) only included around 100 lines. But the type of questions that we are addressing here (fitness as the phenotype) has never been done with these lines. Moreover, note that reference (21) is a review.

>The discussion needs to be modified to explain and elaborate more on the genes mentioned in the results as in why do the researchers think these are universal (PGK1 as potentiator and PFK1, FBA1, TPI1 and PGI1 - line 283). Giving biological reasoning rather than just mentioning they are part of important metabolic pathway as they have done.

There are two elements of the discussion that I think the referee somewhat overlooked: i/The primary outcome of this manuscript is to demonstrate how metabolic enzymes could act as buffers and potentiators of phenotypic variability. Only a few proteins were considered to act as such global modifiers before (see Introduction). Additionally, the only preceding result arguing

jpoyatos@cnb.csic.es

c/ Darwin, 3
Campus de Cantoblanco
28049 MADRID
TEL.: 91 585 5309
FAX: 91 585 4506

that this feature could be a general property of many proteins was demonstrated with a toy network model. ii/ A second important outcome is to show that the very same enzyme could act as buffer or potentiator, as this depends on the specific working regime of metabolism (that can be altered with the nutrient conditions). This emphasizes how working as a modifier represents an intrinsic property of the system generating the phenotype rather than of its constituents. Having said this, I agree with the referee that particular enzymes (e.g., PGK1) appear commonly as modifiers so I added some biological reasoning of why this is so in the discussion.

I thank the reviewer for his/her comment and hope that he/she considers now this manuscript suitable for publication.

jpoyatos@cnb.csic.es

c/ Darwin, 3
Campus de Cantoblanco
28049 MADRID
TEL.: 91 585 5309
FAX: 91 585 4506
